# Design of charge converting lipid nanoparticles via a microfluidic coating technique

**DOI:** 10.1007/s13346-024-01538-5

**Published:** 2024-02-21

**Authors:** Katrin Zöller, Soheil Haddadzadegan, Sera Lindner, Florina Veider, Andreas Bernkop-Schnürch

**Affiliations:** 1 Center for Chemistry and Biomedicine, Department of Pharmaceutical Technology, Institute of Pharmacy, Leopold-Franzens-University of Innsbruck, Innrain 80/82, 6020 Innsbruck, Austria; 2 Thiomatrix Forschungs- und Beratungs GmbH, Research Center Innsbruck, Trientlgasse 65, 6020 Innsbruck, Austria

**Keywords:** Microfluidics, Lipid nanoparticles, Zeta change, DOTAP

## Abstract

It was the aim of this study to design charge converting lipid nanoparticles (LNP) via a microfluidic mixing technique used for the preparation and coating of LNP. LNP consisting of 1,2-distearoyl-sn-glycero-3-phosphocholine (DSPC), cholesterol, N-(carbonyl-methoxypolyethyleneglycol-2000)-1,2-distearoyl-sn-glycero-3-phosphoethanolamine (MPEG-2000-DSPE), and various cationic surfactants were prepared at diverging flow rate ratios (FRR) via microfluidic mixing. Utilizing a second chip in the microfluidic set-up, LNP were coated with polyoxyethylene (9) nonylphenol monophosphate ester (PNPP). LNP were examined for their stability in different physiologically relevant media as well as for hemolytic and cytotoxic effects. Finally, phosphate release and charge conversion of PNPP-coated LNP were evaluated after incubation with alkaline phosphatase and on Caco2-cells. LNP produced at an FRR of 5:1 exhibited a size between 80 and 150 nm and a positive zeta potential. Coating with PNPP within the second chip led to LNP exhibiting a negative zeta potential. After incubation with 1 U/ml alkaline phosphatase for 4 h, zeta potential of the LNP containing 1,2-dioleoyloxy-3-trimethylammonium-propane chloride (DOTAP) as cationic component shifted from − 35 mV to approximately + 5 mV. LNP prepared with other cationic surfactants remained slightly negative after enzymatic phosphate cleavage. Manufacturing of LNP containing PNPP and DOTAP via connection of two chips in a microfluidic instrument proves to show efficient change in zeta potential from negative to positive after incubation with alkaline phosphatase.

**Graphical abstract:**

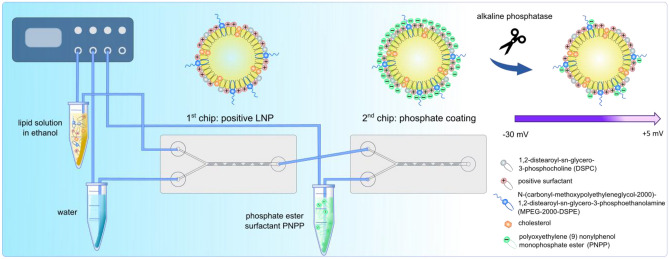

**Supplementary Information:**

The online version contains supplementary material available at 10.1007/s13346-024-01538-5.

## Introduction

Microfluidic preparation of lipid-based nanocarriers such as liposomes [1–3], solid lipid nanoparticles [4–9], or LNP [10, 11] shows many advantages compared to other conventional bulk preparation methods including high reproducibility and precise control over parameters [12–14]. This method is based on condensation of lipids from an organic solution in water. Thereby, fast dilution of the organic phase is essential for the formation of small, monodisperse LNP [10, 11]. Recently, lipid-based nanocarriers produced via microfluidics were mainly intended for the delivery of RNA as in the COVID-19 vaccines, but several research groups have successfully encapsulated also other drugs via microfluidic mixing [5–8, 15–17]. Hydrophobic drugs can be dissolved within the organic solution together with the lipids, whereas hydrophilic drugs are dissolved within the aqueous phase. As a result, microfluidic mixing enables drug loading of lipid-based nanocarriers with either hydrophilic or hydrophobic drugs. Hence, LNP produced via microfluidics could also be of interest for other applications such as oral drug delivery. For oral drug delivery, the main absorption barriers within the gastrointestinal tract are the mucus and the epithelial barrier. Mucus is mainly composed of mucins which are highly glycosylated proteins with sialic and sulfonic acids being negatively charged [18, 19]. Therefore, cationic carriers show strong interactions with mucins resulting in overall low mucus permeability, and only a small amount of particles can reach the absorption membrane. As a result, a negative or neutral surface charge presents advantages in terms of mucus permeation, but once reaching the epithelial cell layer, a cationic surface provides higher cellular uptake. To overcome this so-called polycation dilemma, LNP can be prepared containing phosphate ester surfactants [20], or they can be coated with polyphosphates [18, 19] implementing a negative surface charge. These excipients can be cleaved by alkaline phosphatase, a membrane-bound enzyme within the epithelial cell layer, causing phosphate release and a charge conversion from negative to positive [19, 20]. As a result, LNP first possess a negative surface charge enhancing mucus permeation which is then converted into a positive charge favoring cellular uptake [21].

So far and to best of our knowledge, no charge converting LNP have been produced with a microfluidic device. Therefore, it was the aim of this study to develop zeta potential shifting LNP continuously produced via microfluidics. Consequently, the microfluidic set-up was changed to setting two chips in a row and introducing a third channel for the addition of PNPP. Positively charged LNP were produced in the first chip. These LNP were then transferred to a second chip where they were coated with PNPP to introduce a negative zeta potential using the third channel. Finally, zeta potential and phosphate release were evaluated during incubation with alkaline phosphatase and on Caco2-cells which are able to express the enzyme. Additionally, LNP were investigated for possible cytotoxic and hemolytic effects and for stability in different physiologically relevant media.

## Materials and methods

### Materials

1,2-Distearoyl-sn-glycero-3-phosphocholine (DSPC) was obtained from Avanti Polar Lipids (Alabaster, USA). N-(carbonyl-methoxypolyethyleneglycol-2000)-1,2-distearoyl-sn-glycero-3-phosphoethanol-amine, sodium salt (MPEG-2000-DSPE), and 1,2-dioleoyloxy-3-trimethylammonium-propane chloride (DOTAP) were provided by Lipoid GmbH (Ludwigshafen, Germany) as free samples. MTT (3-(4,5-dimethylthiazol-2-yl)-2,5-diphenyltetrazolium bromide) and cholesterol were purchased from ThermoFisher GmbH (Kandel, Germany). Didecyldimethylammonium bromide was bought from Tokyo Chemical Industry Co. (Tokyo, Japan). Polyoxyethylene (9) nonylphenol monophosphate ester (PNPP, Dextrol™ OC-20) was obtained from Ashland Inc. (Neuhausen, Switzerland) and FaSSIF powder from Biorelevant (London, UK). Tetraheptylammonium bromide (THAB), alkaline phosphatase from bovine intestinal mucosa (10 DEA units/mg solid), and all other chemicals were purchased from Sigma-Aldrich (Vienna, Austria) or other commercial sources and were of analytical grade.

### Preparation and characterization of LNP

A stock solution of 17.7 mM total lipids was prepared by dissolving DSPC, cholesterol, MPEG-2000-DSPE, and the cationic surfactant in molar ratios of 9.4/40/47/1.5 in ethanol. Tetraheptylammonium bromide (THAB), didecyldimethylammonium bromide (DDAB), and DOTAP chloride were utilized as positively charged surfactants. Distilled water was employed as aqueous phase. LNP were fabricated with a herringbone chip with FRR ranging from 2:1, 5:1 and 10:1 (aqueous phase/organic phase) and a total flow rate (TFR) of 3000 µl/min. The flow rate was controlled with a microfluidic instrument (OB1 MK4, Elveflow, Paris, France). Obtained LNP were dialyzed at room temperature for 24 h against distilled water (Spectra-Por^®^ Float-A-Lyzer^®^ 100 kDa, Spectrum, USA). LNP coated with positively charged surfactants were named LNP_THAB_, LNP_DDAB_, and LNP_DOTAP_. Furthermore, LNP were characterized for size, polydispersity index (PDI), and zeta potential with a Zetasizer Nano ZSP (Malvern Panalytical Ltd., United Kingdom) at 37 °C before and after dialysis.

### Coating of LNP with PNPP

**Fig. 1:**
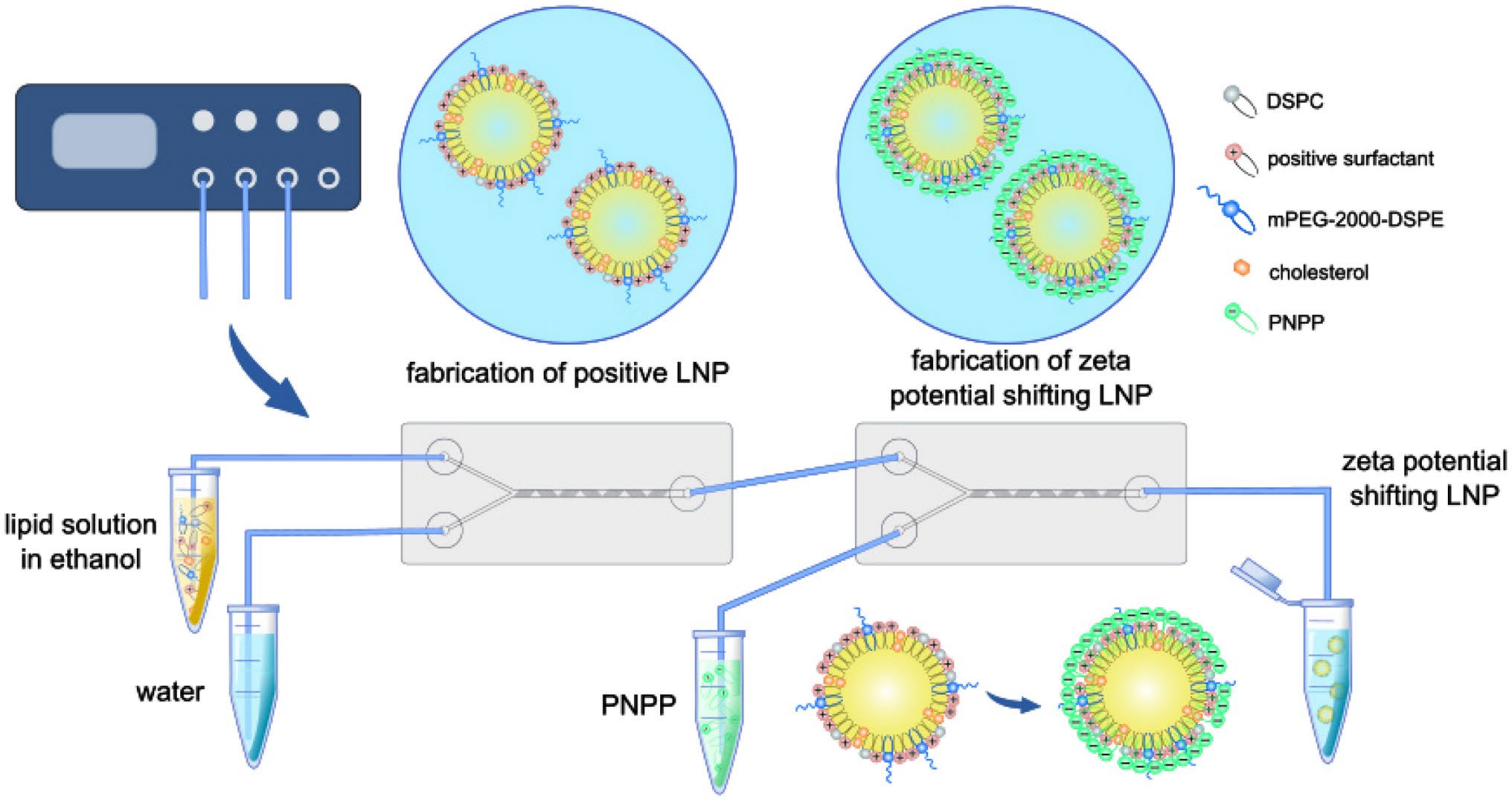
Set-up for the fabrication of zeta potential shifting LNP via microfluidics using two chips

To generate charge converting LNP, positively charged LNP were coated with PNPP being anchored on the surface of these nanoparticles via its lipophilic tail as illustrated in Table 1. Accordingly, two herringbone chips were lined up, and a third channel was introduced in the second chip for the coating with PNPP. The set-up is shown in Fig. 1. LNP with positively charged surfactants were produced in the first chip with an FRR of 5:1 as described above. These positively charged LNP circulated into the second chip where they were coated with a PNPP solution of 5% (m/V) prepared in distilled water at an FRR of 10:1 (LNP:PNPP) and a TFR of 3300 µl/min. Hence, the percentage of PNPP to lipids in the coated LNP was 2.37/1.00 (mol/mol). The obtained LNP coated with PNPP were dialyzed against water at room temperature for 24 h. Moreover, coated LNP were characterized for size, PDI, and zeta potential at 37 °C before and after the dialysis step.

**Table 1:**
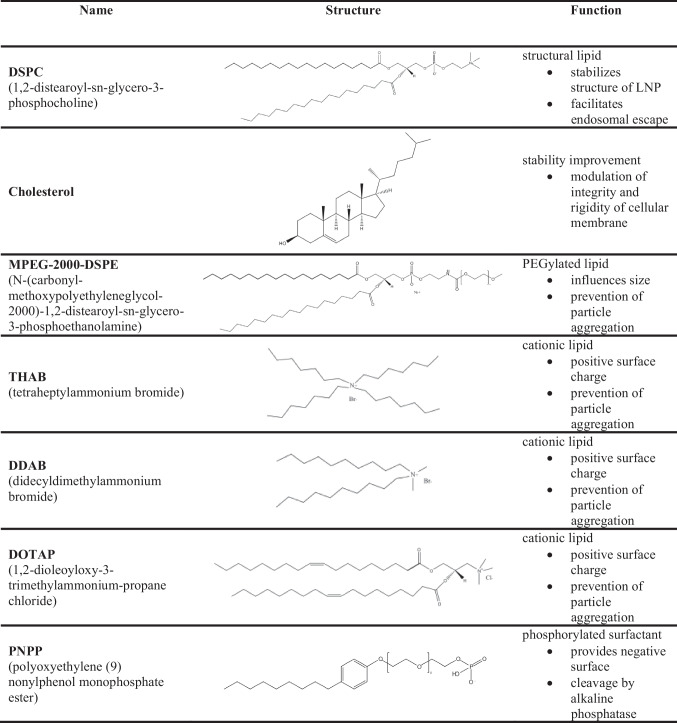
Names, structures and functions of the components within the lipid nanoparticles

### Stability in different media

Dialyzed particles were diluted 1:100 with HEPES buffered saline (HBS), fasted state simulated intestinal fluid (FaSSIF), or fasted state simulated gastric fluid (FaSSGF). HBS consisted of 20 mM HEPES, 1 g/l dextrose, 5 mM KCl, 136.7 mM NaCl, and 1 mM CaCl_2_ and pH was adjusted to 7.4 [22]. FaSSIF and FaSSGF were prepared according to supplier’s manual. Stability of LNP coated with PNPP was also evaluated in 100 mM HEPES pH 7.4 containing 5 mM MgCl_2_ and 0.2 mM ZnCl_2_. The samples were incubated at 37 °C, and size and PDI were determined after 0, 4, and 24 h.

### Cell viability

Possible cytotoxic effects of the LNP were evaluated on Caco2-cells utilizing the MTT assay. The cells were seeded in a density of 20,000 cells per well and incubated for 3 days in minimal essential media (MEM) supplemented with 10% (v/v) heat inactivated fetal calf serum and penicillin/streptomycin solution (100 units/0.1 mg/l) at 37 °C and 95% humidity in an atmosphere of 5% CO_2_ allowing the formation of a monolayer [23]. When starting the assay, MEM was removed, and the cells were washed twice with sterile HBS. And 100 µl of the samples, prepared in sterile HBS, were pipetted on the cells and incubated at 37 °C for 4 h. Triton-X 100 0.1% (v/v) solution and sterile buffer served as positive and negative control for cytotoxic effects, respectively [19]. After 4 h, samples were removed, and cells were washed twice with HBS. A sterile MTT stock solution of 5 mg/ml [24] in HBS was prepared and diluted in a ratio of 1:10 with sterile buffer right before use. Then, 100 µl of diluted MTT solution was applied to cells. The plate was incubated light-protected at 37 °C for 2 h. Thereafter, the solution was removed and formazan crystals were dissolved in 120 µl of dimethyl sulfoxide. The plate was then placed in an orbital shaker incubator at 150 rpm and 37 °C for 10 min to accelerate the dissolution process. And 100 µl of the solution was transferred to a transparent 96-well plate, and absorption was measured at 570 nm [25]. Cell viability is determined according to the following equation:

$$cell\; viability\; \left[\%\right]= \frac{{absorption}_{sample}\mathop{-}{absorption}_{positive \;control}}{{absorption}_{negative\; control}\mathop{-}{absorption}_{positive\; control}}$$

### Hemolysis

Hemolytic activity of LNP and LNP coated with PNPP was evaluated on erythrocytes concentrate which was kindly donated by Tirol Kliniken GmbH (Innsbruck, Austria). The erythrocytes concentrate was suspended in a ratio of 1:200 in sterile HBS for the experiment. LNP were diluted with sterile HBS resulting in concentrations of 1 mM, 0.5 mM, 0.1 mM, and 0.05 mM. HBS served as negative control and 0.5% Triton-X 100 solution as positive control for hemolytic effects. The experiment was started when adding 250 µl of diluted erythrocyte suspension to 250 µl of sample solution, and the samples were incubated in an orbital shaker incubator at 150 rpm and 37 °C for 4 h. Thereafter, samples were centrifuged at 2700 rpm for 10 min, and 100 µl of the supernatant was quantified for released hemoglobin via UV-spectrometry at a wavelength of 415 nm [26]. The extent of hemolysis is determined by the following equation [23]:

$$hemolysis \,\left[\%\right]= \frac{({absorption}_{sample}-{absorption}_{negative})}{({absorption}_{positive}-{absorption}_{negative})}\times 100$$

### Mucus diffusion study

The Transwell method for mucus diffusion was utilized for this study as described by Friedl et al. [27] with some modifications. A 24-well Transwell plate was equipped with thin cert inserts possessing a pore size of 3 µm and a surface area of 33.6 cm^2^ (Greiner Bio-One, Germany). Each insert was covered with 60 mg of porcine intestinal mucus, and the plate was shaken at 1200 rpm and 37 °C for 5 min on a thermomixer in order to obtain a homogenous layer. Prior, the mucus was collected from a freshly excised porcine intestine, and it was purified according to an established protocol [28]. For the samples, LNP and PNPP-coated LNP were loaded with 0.1% coumarin 6. Therefore, 85 µl of a 1 mg/ml ethanolic solution of coumarin 6 was added to the lipid stock solution, and LNP were prepared as described above. Coumarin 6-labeled LNP and LNP coated with PNPP were diluted with HBS to a concentration of 1 mM, and 300 µl of this solution was pipetted into the donor chamber of the plate. The acceptor chamber contained 600 µl of HBS. After application of the samples, the plate was incubated at 37 °C and 80 rpm in a shaking incubator. Aliquots of 100 µl were withdrawn from the acceptor chamber each hour up to 4 h and replaced by 100 µl of fresh preheated HBS. For the 100% control value, the same experiment was conducted without mucus to evaluate the exact amount of labelled LNP that can penetrate the membrane. The samples were analyzed for fluorescence intensity at an excitation wavelength of 457 nm and an emission wavelength 501 nm of with a microplate reader. The amount of permeated LNP was calculated cumulative referring to the 100% control value.

### Enzyme-induced charge conversion

Dialyzed LNP coated with PNPP were diluted 1:1 (v/v) with 100 mM HEPES pH 7.4 containing 5 mM MgCl_2_ and 0.2 mM ZnCl_2_. Enzyme solution was prepared freshly before each experiment. Hence, alkaline phosphatase was dissolved in buffer and added to the samples to obtain a final concentration of 1 U/ml. A 1 ml of each samples was filled in a dialysis tube (Spectra-Por^®^ Float-A-Lyzer^®^ 300 kDa, Spectrum, USA) which were put in 50-ml falcon tubes containing 30 ml HEPES buffer containing alkaline phosphatase in a concentration of 1 U/ml. The samples were incubated at 37 °C in an orbital shaker incubator (Orbital Shaker Incubator ES-80, Royston, Grant Instruments Ltd, UK) for 4 h. After that time period, zeta potential was evaluated with the Zetasizer utilizing a dip cell. Samples without enzyme addition served as control.

### Enzyme-induced phosphate release

The malachite green assay was used to determine the amount of released monophosphates during incubation with alkaline phosphatase. Firstly, 500 µl of LNP coated with PNPP were diluted with 500 µl of 100 mM HEPES pH 7.4 containing 5 mM MgCl_2_ and 0.2 mM ZnCl_2_. The reaction was started by addition of alkaline phosphatase solution resulting in a final concentration of 1 U/ml. The samples were incubated in a Thermomixer (Eppendorf ThermoMixer^®^ C, Eppendorf AG, Hamburg, Germany) at 37 °C and 500 rpm. At predetermined time points (0, 30, 60, 120, 180, and 240 min), 50 µl of the samples were transferred to a transparent 96-well plate, and the reaction was stopped by addition of 5 µl of 3.6 M H_2_SO_4_. Malachite green reagent was prepared by dissolving 15 mg of malachite green in 10 ml 3.6 M H_2_SO_4_ and 0.4 ml Triton-X 100 11% (m/v). The mixture was stirred for 20 min at room temperature. Afterwards, 6 ml of ammonium molybdate 8% (m/v) were added dropwise under constant stirring. Then, 100 µl of the reagent was added to the samples, and absorbance was measured at 630 nm with a microplate reader (Tecan Spark, Tecan Group Ltd., Männedorf, Switzerland). A calibration curve with KH_2_PO_4_ was generated in order to calculate the amount of released monophosphate [20]. LNP without alkaline phosphatase incubated under the same conditions served as control.

### Phosphate release on Caco2 cells

Caco2-cells were seeded in 24 well plates in a density of 25 000 cells per well and incubated in MEM containing 10% (v/v) heat inactivated fetal calf serum and penicillin/streptomycin solution (100 units/0.1 mg/l) at 37 °C and 95% humidity in an atmosphere of 5% CO_2_ for 10 days. MEM was exchanged every second day [20]. Before application of the samples, cells were washed twice with sterile HBS. And 500 µl of LNP coated with PNPP in a concentration of 0.05 mM prepared in sterile HBS were added to the cells and incubated for 4 h at 37 °C. Aliquots of 50 µl were withdrawn after 30, 60, 120, 180, and 240 min and transferred to a 96-well plate where the reaction was stopped by the addition of 5 µl of 3.6 M H_2_SO_4_. As a control, the same experiment was conducted in presence of phosphatase inhibitor cocktail II (PIC2) 1% (v/v). Prior to the experiment, cells were incubated with HBS containing 1% PIC2 for 60 min before application of the samples [18, 19]. Released phosphate was quantified via the malachite green assay as described above.

### Statistical design and analysis of data

All experiments were performed at least in triplicates and results were presented as means ± standard deviation. Statistical analysis was performed via one-way or two-way ANOVA (GraphPad Prism 5) with *p* < *0.05* considered level of significance.

## Results

### Preparation and characterization of LNP

**Fig. 2:**
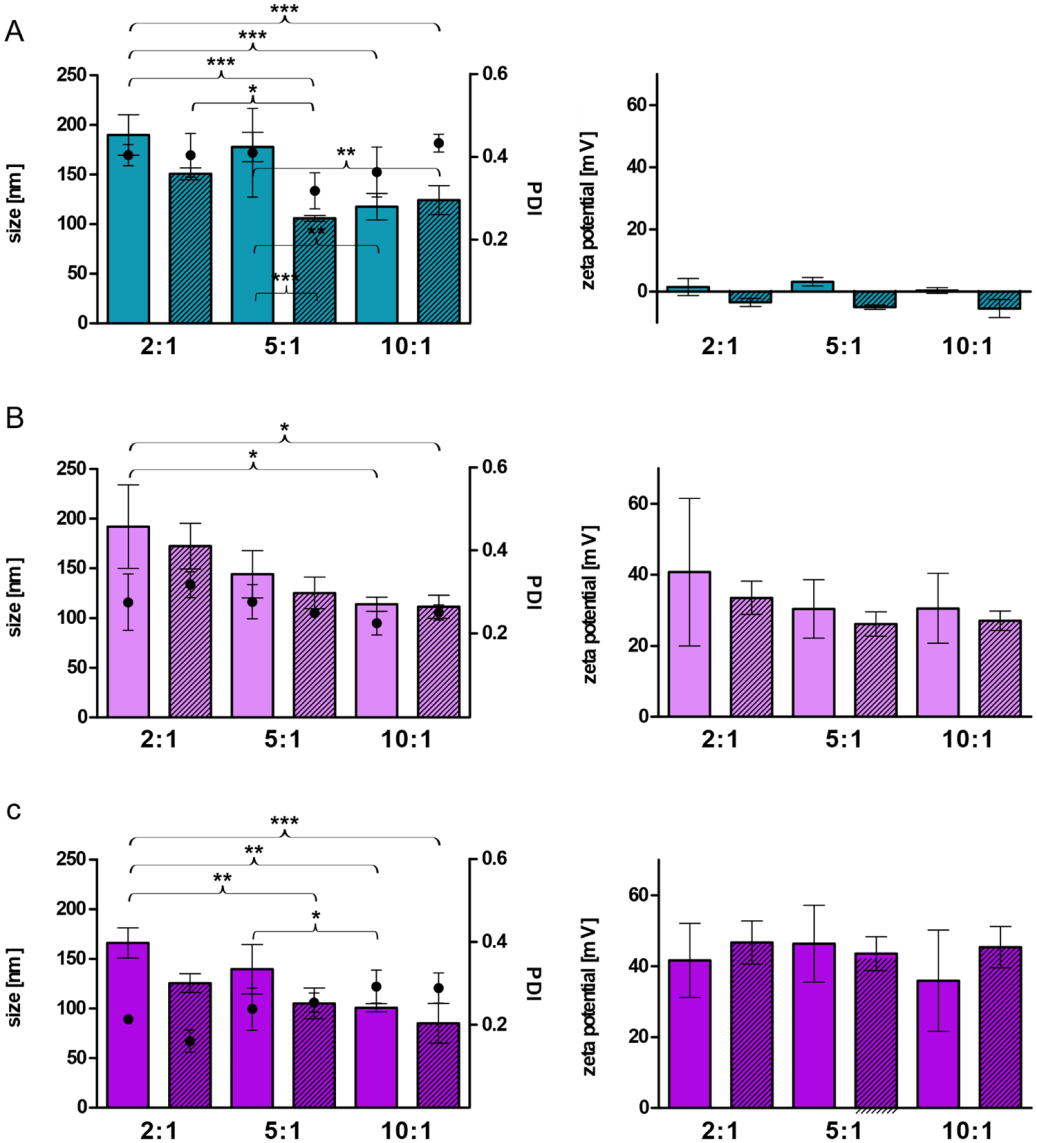
Size (bars) [nm], PDI (points), and zeta potential [mV] of LNP containing positively charged surfactants produced with varying FRR before (plain bars) and after dialysis (streaked bars) against distilled water. **A** LNP_THAB_; **B** LNP_DDAB_; **C** LNP_DOTAP_. Data are presented as means ± sd. Significant differences are indicated as *p < 0.05; **p < 0.01; ***p < 0.001

LNP with positively charged surfactants were prepared with varying FRR as shown in Fig. 2. LNP fabricated via microfluidics with cationic surfactants were so far mainly intended for the encapsulation of RNA [10, 11, 29–31], and it is known that size and PDI decrease when the flow rate of the aqueous phase is increased [1, 32, 33]. Similar results were found within our study. Especially, increasing the FRR from 2:1 to 5:1 or 10:1 caused the formation of smaller LNP. On the other hand, there were only slight differences between LNP produced at an FRR of 5:1 and LNP manufactured at 10:1. Hence, an FRR of 5:1 was used to generate LNP for all further experiments. Generally, all particles formed at an FRR of 5:1 exhibited a size between 140 and 90 nm and PDI below 0.4 after the dialysis step. Obviously, LNP containing positively charged surfactants exhibited a positive zeta potential. Interestingly, LNP_THAB_ were more or less neutral although THAB was positively charged.

**Fig. 3:**
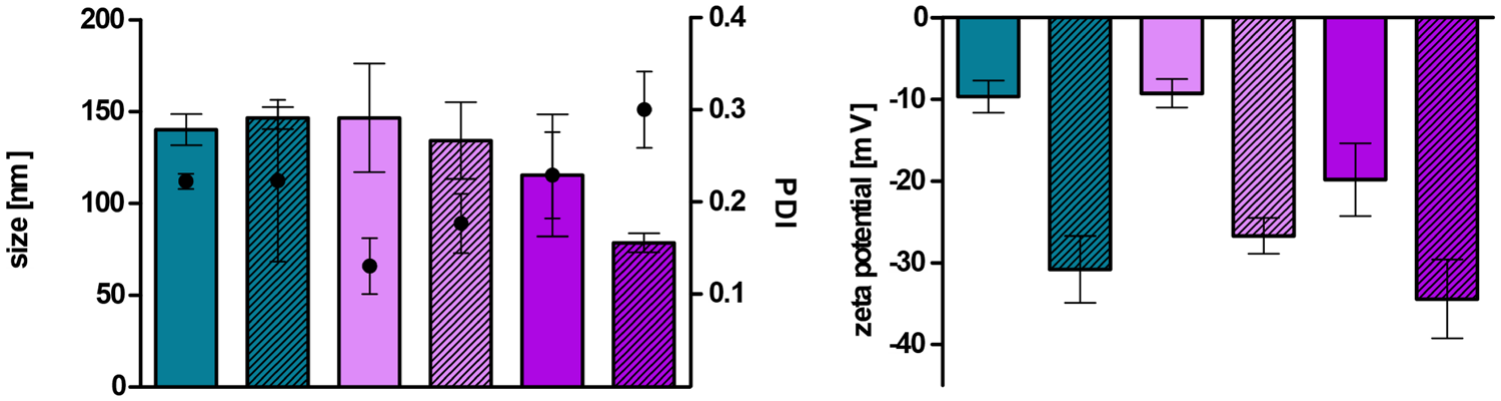
Size [nm] (bars), PDI (points), and zeta potential [mV] of LNP coated with PNPP before and after dialysis. LNP before dialysis are indicated as blank bars and LNP after dialysis as streaked bars. LNP are indicated as follows: LNP_THAB_ (turquoise), LNP_DDAB_ (light pink), and LNP_DOTAP_ (pink). Data are presented as means ± sd

Development of charge converting LNP can be derived either via incorporation of phosphorylated surfactants and polymers into the lipid-based nanocarriers or via coating with polyphosphates [19]. In our study, the phosphate ester surfactant PNPP was added online in a second chip on the positively charged LNP fabricated in the first chip. The study from Akkus-Dagdeviren et al. [20] compared different phosphate bearing surfactants regarding their ability to form charge reversal self-emulsifying drug delivery systems (SEDDS). They found highest phosphate release and zeta potential shift for SEDDS fabricated with PNPP which was the reason why PNPP was chosen for the purpose of our study. After coating with PNPP within the second chip, a negative zeta potential was obtained, and sizes of the particles were around 150 nm with PDIs below 0.4 as shown in Fig. 3. Coated LNP_DOTAP_ even exhibited a size below 100 nm after dialysis.

**Fig. 4:**
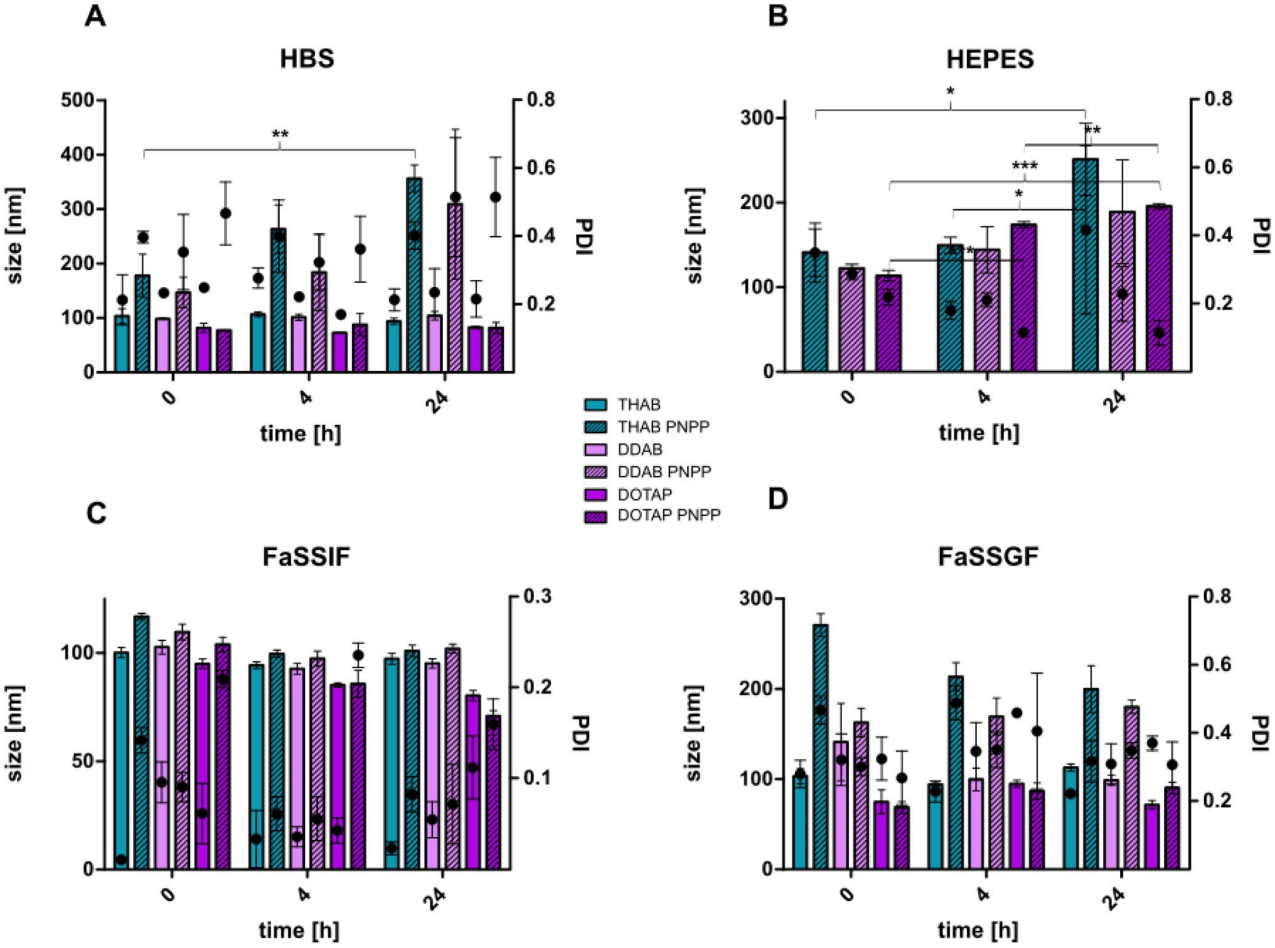
Alterations in size [nm] (bars) and PDI (points) of LNP and PNPP-coated LNP during incubation in HBS (**A**), 100 mM HEPES containing 5 mM MgCl_2_ and 0.2 mM ZnCl_2_ (**B**), FaSSIF (**C**), and FaSSGF (**D**) at 37 °C. LNP are indicated as follows: LNP_THAB_ (turquoise), LNP_DDAB_ (light pink), and LNP_DOTAP_ (pink). LNP without PNPP are shown as blank bars, whereas LNP coated with PNPP are depicted as streaked bars. Data are presented as means ± sd. Significant differences are indicated as *p < 0.05; **p < 0.01; and ***p < 0.001

LNP were furthermore evaluated for their stability in different physiologically relevant media (Fig. 4). All particles were stable over a time period of 24 h within FaSSIF and FaSSGF. LNP produced without PNPP did not show any significant increase in size and PDI in the tested media. Up to 4 h, all LNP coated with PNPP did not exceed 200 nm, indicating that the particles remained stable for the duration of the following experiments. Nevertheless, PNPP-coated LNP containing THAB and DDAB grew to almost 400 nm in HBS after 24 h and furthermore, PDI was accelerating for all PNPP-coated LNP and all coated LNP grew to around 200 nm in HEPES after 24 h.

### Cytotoxicity and hemolysis

**Fig. 5:**
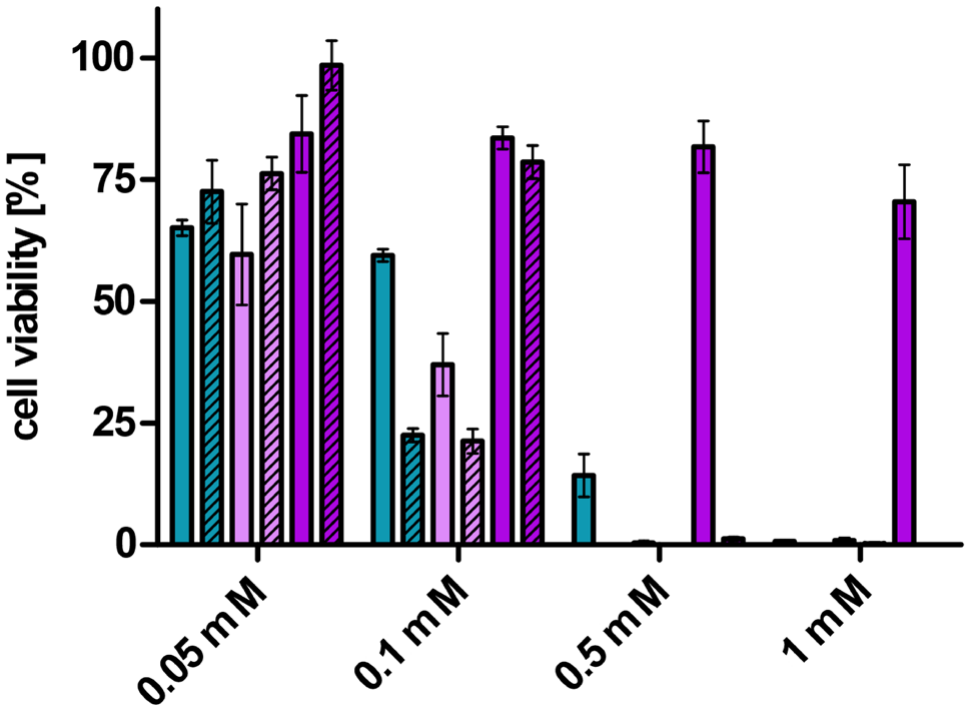
Cell viability [%] of Caco2-cells after incubation with samples at 37 °C for 4 h. LNP are indicated as follows: LNP_THAB_ (turquoise), LNP_DDAB_ (light pink), and LNP_DOTAP_ (pink). LNP without PNPP are presented as blank bars and PNPP-coated LNP as streaked bars. Data are presented as means ± sd

**Fig. 6:**
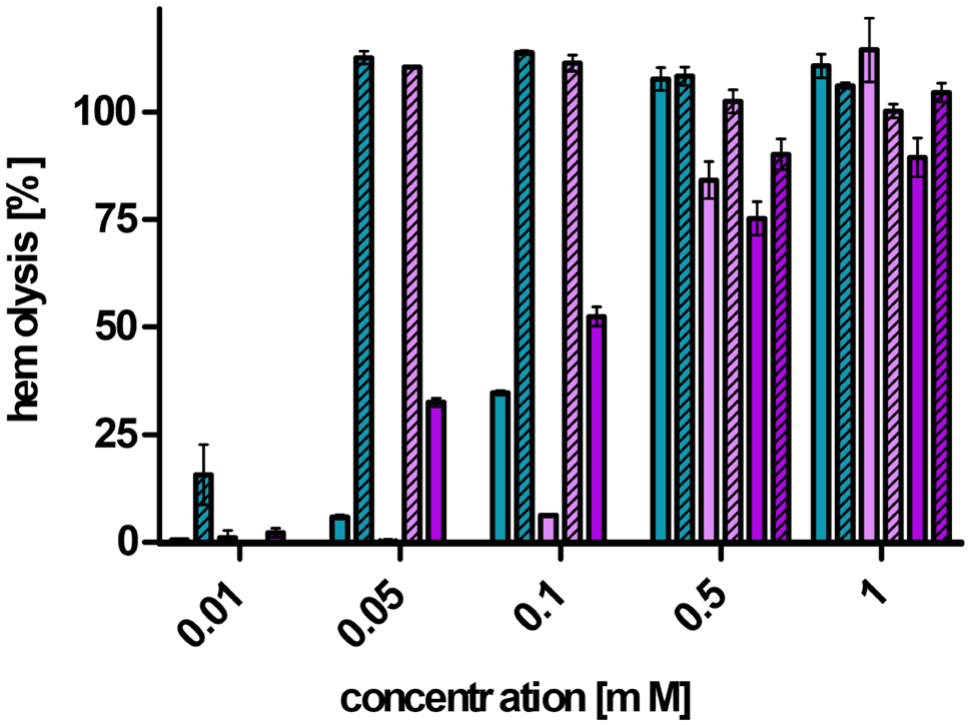
Hemolysis [%] of erythrocytes after incubation with LNP and PNPP-coated LNP in increasing concentrations at 37 °C for 4 h. LNP are indicated as follows: LNP_THAB_ (turquoise), LNP_DDAB_ (light pink), and LNP_DOTAP_ (pink). LNP without PNPP are presented as blank bars and LNP coated with PNPP as streaked bars. Data are presented as means ± sd

Potential cytotoxic effects were evaluated with the MTT assay (Fig. 5). Formulations are generally considered safe when they exhibit a cell viability above 80% [34]. Therefore, LNP with THAB and DDAB were more toxic than LNP containing DOTAP. Hence, LNP_DOTAP_ were only toxic at the highest tested concentration of 1 mM. DOTAP is a cationic lipid often used for liposomal transfection [35], and it shows less toxicity than other cationic lipids [36]. For instance, Filion et al. [37] prepared liposomes with dioleoylphosphatidylethanolamine (DOPE) and DOTAP or dimethyldioctadecylammonium bromide (DDOAB) as cationic compounds. Liposomes DOPE/DDOAB were more toxic than liposomes of DOPE/DOTAP. Furthermore, LNP coated with PNPP showed increased toxicity compared to their corresponding LNP. Comparable results were found within the hemolysis experiment as illustrated in Fig. 6 where PNPP-coated LNP exhibited higher hemolytic activity than the LNP without PNPP. Generally, hemolysis studies can either be used for evaluation of toxicity or for the prediction of membrane interaction [19, 23]. LNP_DOTAP_ expressed slowly accelerating hemolytic activity with increasing concentrations, whereas LNP_DDAB_ and LNP_THAB_ showed 100% hemolysis at concentrations of 0.5 mM and 1 mM with only minor hemolytic activity below these concentrations. DOTAP is known to show fusogenic properties which could explain the slowly accelerating hemolysis as it interacted with the membrane at all tested concentrations without showing complete disruption. When incorporating PNPP into LNP_DOTAP_, the slowly accelerating trend vanished and suddenly complete hemolysis was detected at a concentration of 0.5 mM. After the coating step, PNPP was the preliminary surface compound on the nanoparticles also altering their properties.

### Charge converting LNP

**Fig. 7:**
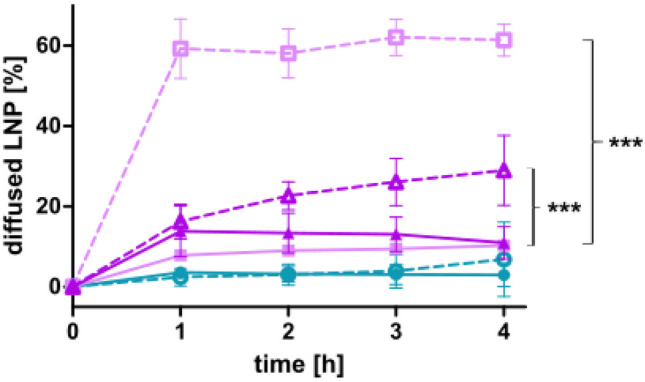
Cumulative diffusion of LNP [%] labeled with coumarin 6 through porcine intestinal mucus over a time period of 4 h. LNP are indicated as follows: LNP_THAB_ (turquoise), LNP_DDAB_ (light pink), and LNP_DOTAP_ (pink). PNPP-coated LNP are shown as dotted lines and unfilled symbols, whereas LNP without PNPP are presented as fully drawn lines and filled symbols. Data are presented as means ± sd. Significant differences are indicated as *p < 0.05, **p < 0.01, and ***p < 0.001

**Fig. 8:**
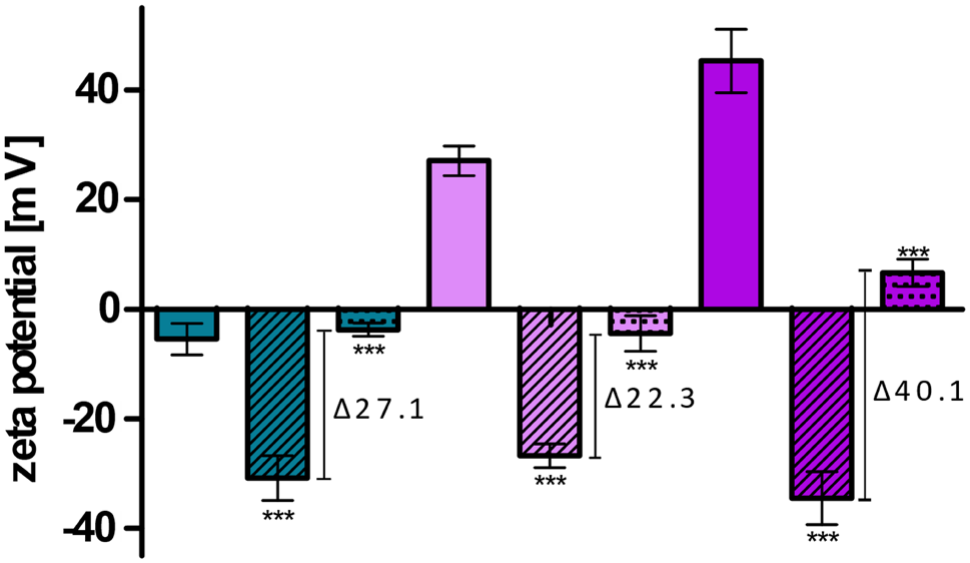
Zeta potential [mV] shift of PNPP-coated LNP. Zeta potential of LNP without PNPP are shown as blank bars, LNP coated with PNPP as streaked bars, and LNP after incubation with alkaline phosphatase for 4 h as dotted bars. LNP are indicated as follows: LNP_THAB_ (turquoise), LNP_DDAB_ (light pink), and LNP_DOTAP_ (pink). Data are presented as means ± sd. Significant differences are indicated as *p < 0.05, **p < 0.01, and ***p < 0.001

**Fig. 9:**
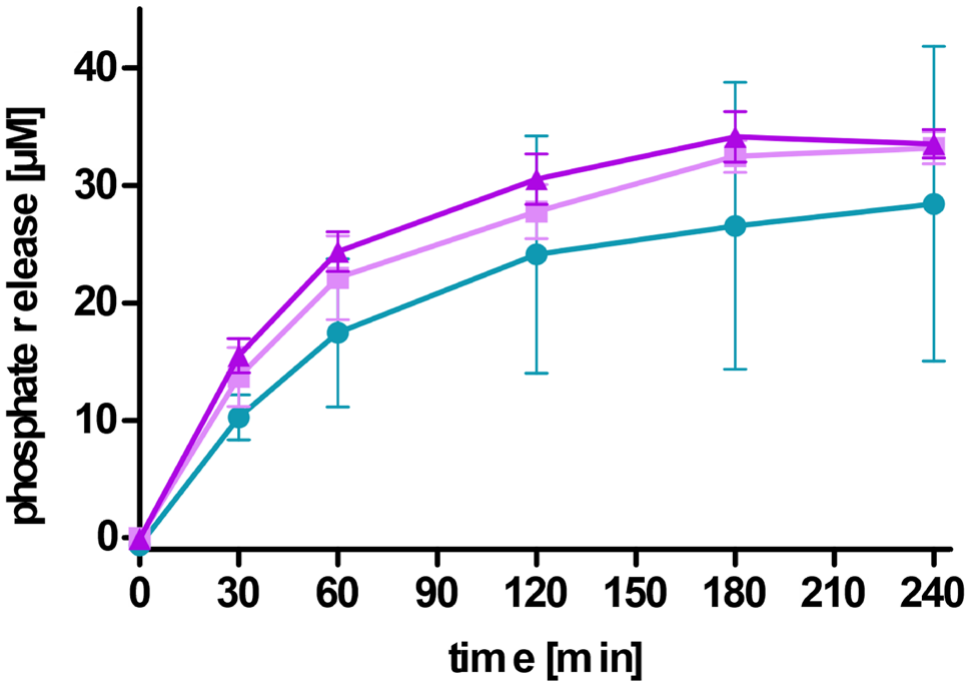
Phosphate release [µM] of PNPP-coated LNP during incubation with 1 U/ml alkaline phosphatase at 37 °C for 4 h. LNP are indicated as follows: LNP_THAB_ (turquoise), LNP_DDAB_ (light pink), and LNP_DOTAP_ (pink). Data are presented as means ± sd. Significant differences are indicated as *p < 0.05, **p < 0.01, and ***p < 0.001

**Fig. 10:**
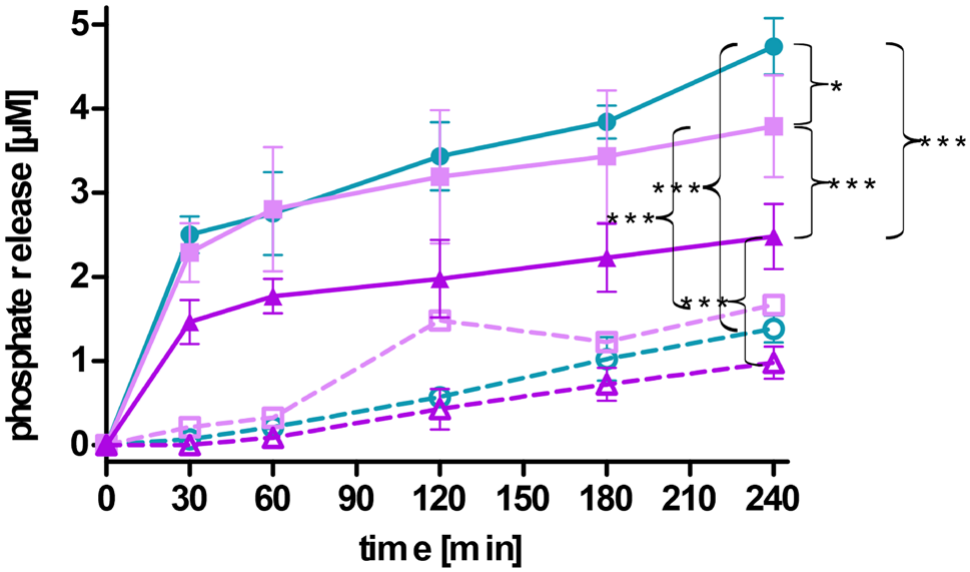
Phosphate release [µM] of LNP coated with PNPP during incubation on Caco2-cells at 37 °C for 4 h. LNP are indicated as follows: LNP_THAB_ (turquoise), LNP_DDAB_ (light pink), and LNP_DOTAP_ (pink). Samples with PIC2 are presented as dotted lines, whereas samples without the inhibitor are shown as continuous lines. Data are presented as means ± sd. Significant differences are indicated as *p < 0.05, **p < 0.01; ***p < 0.001

Mucosal tissue covers the gastrointestinal tract and acts as a barrier above the epithelial cell layer [19, 27]. In order to reach this epithelial cell layer, LNP have to permeate through the mucus layer. As investigated on porcine intestinal mucus and presented in Fig. 7, PNPP-coated LNP_DDAB_ and LNP_DOTAP_ showed higher mucus diffusion over a time period of 4 h compared to their corresponding LNP without PNPP. Moreover, LNP_THAB_ did not present this ability and revealed the lowest diffusion. Comparable results were found in another study where negatively coated lipid-based nanocarriers correlated to higher mucus diffusion and positively charged carries to lower diffusion [19]. In theory, after permeation through this mucus layer, the formulation reaches the epithelial cell layer and phosphate release, and charge conversion is mediated via alkaline phosphatase. This enzyme catalyzes the hydrolysis of phosphate monoesters causing the release of monophosphates. As a result, the amount of negatively charged groups on the surface of the LNP is reduced which causes a shift in zeta potential [21]. Mammalian alkaline phosphatases possess one magnesium ion and two zinc ions on their active site which enable the enzymatic activity [18]. Hence, experiments were conducted in HEPES buffer supplemented with MgCl_2_ and ZNCl_2_. Figure 8 presents the zeta potential of LNP before and after addition of PNPP and after incubation with alkaline phosphatase. As a result, the zeta potential shifts back to + 5 mV for LNP produced with DOTAP and to almost neutral for LNP produced with DDAB and THAB after enzymatic incubation due to the release of monophosphates. This phosphate release is depicted in Figs. 9 and 10. Since also LNP_THAB_ did not exhibit a positive zeta potential before the addition of PNPP, no shift to positive values could be expected. Additionally, phosphate release induced by the isolated enzyme correlated with the shift in zeta potential: LNP_DOTAP_ showed the highest release of phosphate, and furthermore it showed the highest zeta potential shift and managed to convert from negative to positive. Generally, phosphate release reached a plateau after approximately 180 min which might be attributed to the exhaustive amount of released monophosphate inhibiting the enzyme [18, 38]. The controls without enzyme addition showed no phosphate release and no significant shifts in zeta potential (data not shown). Alkaline phosphatase is also expressed by Caco2-cells in a level similar to the human intestine [19, 20, 39]. When the cells have grown in a confluent monolayer, highest enzymatic activity can be detected [40]. Hence, cells were grown for at least 10 days in order to ensure formation of a monolayer. All LNP coated with PNPP showed phosphate release when applied on Caco2-cells. Interestingly, LNP_THAB_ showed two times higher phosphate release than LNP_DOTAP_ which differs from the results found during incubation with the enzyme itself. As a control, PIC2 was added to the samples inhibiting the membrane bound enzyme and significantly lower phosphate release was detected which is in confirmation with other studies [19, 20, 41].

## Discussion

Fabricating LNP via microfluidics bears several advantages including precise control over parameters, high reproducibility, and the possibility for industrial scale-up [10, 11]. Properties of LNP can be affected by various factors, including FRR, TFR, dimensions of the chip, lipid composition, and buffer choice [32, 42]. The success of this method was further proven with the admission of the COVID-19 vaccines [10, 42]. Besides the delivery of nucleic acids, those lipid-based nanocarriers including liposomes, self-emulsifying drug delivery systems (SEDDS) and LNP can be promising formulations for oral drug delivery as well. Nevertheless, these systems should stay physically stable, meaning the particles should maintain a homogenous size distribution and prevent aggregation which could further cause phase separation and escape of the encapsulated drug [43]. Furthermore, the formulation can be impacted by lipid degradation either during storage or within the gastrointestinal tract. Especially changes in pH from acidic conditions in the stomach to neutral or alkaline environments in the intestine, variable concentration of salts, and electrolytes and gastrointestinal enzymes like pancreatic lipase are able to degrade those lipid-based nanocarriers, and continuing decomposition could as well lead to undesired drug release [44–46]. Possible strategies to overcome these problems can be based on suitable surface coating, e.g., PEG which sterically stabilizes the particles preventing aggregation and also hampers the adsorption of enzymes [45, 47]. Moreover, non-digestible components can be chosen for the composition of the nanoparticles. LNP in our study were based on cholesterol, DSPC as structural lipid, MPEG-2000-DSPE as PEGylated lipid, and positive surfactants providing a positive surface charge of LNP. Cholesterol can improve the stability of LNP by modulating the integrity and rigidity of the cellular membrane [11, 42, 48, 49]. DSPC is able to form a lamellar phase which stabilizes the structure of LNP and furthermore facilitates endosomal escape [11, 48]. The PEGylated lipid mostly affects size and further prevents particle aggregation due to sterical hindrance [11, 42, 48–50]. Since cationic surfactants provide a positive zeta potential of LNP, they also contribute to prevent particle aggregation via electrostatic repulsive forces [50]. LNP_THAB_ did not show a positive zeta potential although THAB is permanently positively charged due to the quarternary ammonium structure. It is possible that the charge of THAB was shielded by the PEGylated surfactant. In stability studies, LNP_THAB_ was less stable in HBS and HEPES compared to the other nanoparticles as it might show higher interparticle interactions than the particles with DDAB and DOTAP. Additionally, due to the presence of salts within HBS and HEPES, these cations could interact with the phosphate groups of the surfactant, therefore masking their charge and decreasing electrostatic repulsion which might be an explanation for the increase in size and PDI of PNPP-coated LNP.

Furthermore, both size and surface charge have an impact on cytotoxicity. As it was shown that liposomes of DOPE/DDOAB were more toxic than liposomes of DOPE/DOTAP [37], the cationic components THAB and DDAB used within our study are structurally comparable to DDOAB, as they consist of a quarternary ammonium substructure supplemented with longer alkyl chains which might explain the higher toxicity of LNP containing THAB and DDAB in comparison to LNP_DOTAP_. Moreover, positively charged LNP show higher interactions with cells due to the presence of negatively charged phospholipids and proteins within the cellular membrane [50, 51]. Nevertheless, cationic LNP without PNPP were less toxic than PNPP-coated LNP which exhibit a negative surface charge. Since LNP coated with PNPP contained higher amounts of surfactants after coating with PNPP, this could have caused membrane disruption which also correlates with the results obtained in the hemolysis experiment. Additionally, interactions between the carriers and erythrocytes are reduced when PEG chains of mPEG-2000-DSPE are presented on the surface of the particles [52]. PNPP-coated LNP might thus show a higher hemolytic activity since PNPP can overcome the PEG corona with its spacer and thus can initiate interactions between LNP and erythrocytes. Furthermore, slowly accelerating hemolytic activity was detected for LNP_DOTAP_, whereas the other nanoparticles suddenly showed a high increase in hemolysis at a specific concentration. This might be attributed to different types of membrane interaction of the nanoparticles mediated by the different cationic surfactants and PNPP. For instance, Kitagawa et al. [53] prepared vesicles consisting of DOTAP and several phosphatidylcholines. Their vesicles interacted with the erythrocyte membrane changing their surface charge and fluidity causing interactions among erythrocytes and some fusogenic events. The accelerating hemolytic activity changed also into full hemolysis at a certain concentration when LNP_DOTAP_ were coated with PNPP.

Preparation of charge converting lipid-based nanocarriers can either be achieved via coating with phosphorylated surfactants or with polyphosphates. Akkus-Dagdeviren et al. [20] developed SEDDS with different phosphorylated surfactants, and Knoll et al. [19] coated nanostructured lipid carriers (NLC) with polyphosphates. Both, SEDDS and NLC, showed charge conversion after incubation with alkaline phosphatase. In our study, we coated the positively charged LNP with a phosphate ester surfactant during the manufacturing step which enabled a charge conversion. To the best of our knowledge, this is the first time that charge conversion within a microfluidic instrument is reported when combining two chips. This charge conversion might be beneficial in terms of mucus permeating properties and cellular uptake as shown in other studies [19–21]. Mucus permeation is facilitated by negative surface charges, and also PEG increases particle surface hydrophilicity and thus hampers interactions with mucus [21]. Especially the coating of LNP_DDAB_ and LNP_DOTAP_ with negatively charged PNPP resulted in higher mucus diffusion thus proving this concept. Since LNP_THAB_ possessed a neutral charge before the coating step, no significant difference was detected in terms of mucus diffusion after the coating. Nevertheless, for LNP prepared with DDAB and DOTAP, this coating improved the mucodiffusive properties, and once reaching the epithelial cell layer and after enzymatically-mediated charge conversion, the resulting positive surface charge enhances internalization. LNP with DDAB and THAB showed only a shift towards slightly negative values after incubation with alkaline phosphatase which might be caused by the shielding effect of the PEGylated lipid [23]. This PEG-corona masks the positive charges of the LNP and results in a lower zeta potential [21]. Comparable results were found by Akkus-Dagedviren et al. [20] where only PEG-containing SEDDS with phosphorylated surfactants bearing a polyethoxylated spacer resulted in a positive zeta potential after enzymatic cleavage. This linker enabled accessibility of the enzyme towards the phosphate group since it was heading out of the PEG corona. Additionally, MPEG-2000-DSPE is longer than PNPP and can thus hamper the access of alkaline phosphatase which results in lower phosphate cleavage and less change in zeta potential near positive environment. Merely LNP containing DOTAP provided a charge conversion from approximately – 35 to + 5 mV. Therefore, charge conversion of LNP seems to depend strongly on the cationic component. DDAB and THAB are on the one hand both quarternary ammonium compounds branched with alkyl chains. DOTAP on the other hand is a cationic lipid often utilized for the preparation of cationic liposomes or liposome-DNA-complexes promoting fusion with the cellular membrane [36]. Moreover, DOTAP has a conical shape [54] which might be beneficial for the structural confirmation of the LNP and making it more accessible for the enzyme. Still, a shift back to the original charge of the nanoparticles without PNPP was not feasible since cleaved free monophosphate can accumulate and interact with the positively charged quarternary ammonium groups of the surfactant. Comparable results were presented by Knoll et al. [19]. In presence of PIC2, only low amounts of phosphate were released indicating that PIC2 was unable to completely inhibit all phosphatases expressed by Caco2. Taken all, our continuous microfluidic mixing method provides a sound solution for the preparation of charge converting LNP.

## Conclusion

This is the first time reported that charge converting LNP were produced via microfluidic mixing. The set-up consisted of two herringbone chips where the positively charged LNP were produced within the first chip and afterwards they were coated with a negatively charged phosphate ester surfactant. LNP exhibited sizes around 100–200 nm with a PDI below 0.4. In the toxicity and hemolysis studies, LNP containing THAB and DDAB proved to be more toxic than LNP_DOTAP_ which showed lowest toxicity and favorable hemolytic activity correlating to endosomal escape. After the coating, LNP exhibited higher toxicity and hemolysis due to the higher amount of surfactant within the nanoparticles. Incubation with alkaline phosphatase led to a shift in zeta potential towards almost neutral environment for LNP containing DDAB and THAB. A shift to + 5 mV was observed with DOTAP. Phosphate release during incubation with the isolated enzyme and on Caco2-cells proved the successful cleavage of the phosphate ester. Therefore, fabrication of LNP containing DOTAP and PNPP led to a sufficient zeta potential change after enzymatic cleavage of PNPP.

## Supplementary Information

Below is the link to the electronic supplementary material.

Supplementary file1 (DOCX 153 KB)

## Data Availability

The authors confirm that the data supporting the findings of this study are available within the article.
