# Supplementary material for: Design of charge converting lipid nanoparticles via a microfluidic coating technique
Source: Drug Deliv Transl Res. 2024 Feb 21;14(11):3173–85. doi: 10.1007/s13346-024-01538-5 (PMC11445316; doi:10.1007/s13346-024-01538-5)
Supplement: Supplementary file 1 — Supplementary file1 (DOCX 153 KB) [file 13346_2024_1538_MOESM1_ESM.docx]

Supplementary data

# Phosphate release on HeLa cells

HeLa were seeded in a density of 50 000 cells/well in a 24-well plate. Cells were incubated in Dulbecco's Modified Eagle Medium (DMEM) containing 10% (v/v) heat inactivated fetal calf serum and penicillin/streptomycin solution (100 units/0.1 mg/l) at 37°C and 95% humidity in an atmosphere of 5% CO_2_ for 10 days. DMEM was exchanged every second day. Before application of the samples, cells were washed twice with sterile HBS. 500 µl of LNP coated with PNPP in a concentration of 0.05 mM prepared in sterile HBS were added to the cells and incubated for 4 h at 37°C. Aliquots of 50 µl were withdrawn after 30, 60, 120, 180 and 240 min and transferred to a 96-well plate where the reaction was stopped by the addition of 5 µl of 3.6 M H_2_SO_4_ [1, 2]. Released phosphate was quantified via the malachite green assay. For this assay, malachite green reagent was prepared by dissolving 15 mg of malachite green in 10 ml of 3.6 M H_2_SO_4_ and 0.4 ml of Triton-X 100 11% (m/v). The mixture was stirred for 20 min at room temperature. Afterwards, 6 ml of ammonium molybdate 8% (m/v) were added dropwise under constant stirring. 100 µl of the reagent was added to the samples and absorbance was measured at 630 nm with a microplate reader. A calibration curve with KH_2_PO_4_ was generated in order to calculate the amount of released monophosphate [3].


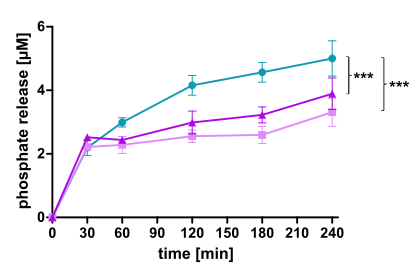


**Figure S1:** Phosphate release [µM] of LNP coated with PNPP during incubation on HeLa cells at 37°C for 4 h. LNP are indicated as follows: LNP_THAB_ (turquoise), LNP_DDAB_ (light pink) and LNP_DOTAP_ (pink). Data are presented as means ± sd. Significant differences are indicated as * p < 0.05; ** p < 0.01; *** p < 0.001.

HeLa cells are an epithelial cell line able to express alkaline phosphatase [4, 5]. Hence, phosphate release from the coated LNP could be monitored during the incubation. The obtained results where comparable to the results found on Caco2-cells. Highest phosphate release was achieved by LNP_THAB_ but there was no significant difference between LNP_DDAB_ and LNP_DOTAP_.

# Cellular uptake

Cellular uptake studies were conducted as described by Knoll et al. [2] with some minor modifications. Therefore, Caco2-cells were seeded in a concentration of 25 000 cells/well and grown for 10 days in minimal essential media (MEM) supplemented with 10% (v/v) heat inactivated fetal calf serum and penicillin/streptomycin solution (100 units/0.1 mg/l) at 37°C and 95% humidity in an atmosphere of 5% CO_2_ until they reached 100% confluency. When starting the experiment, cells were washed twice with sterile HBS before application of coumarin 6-labeled LNP coated with PNPP in a concentration of 0.05 mM prepared in HBS. The same experiment was conducted in presence of PIC2 1% (v/v). After 3 h of incubation at 37°C, samples were withdrawn and cells were washed twice with cold buffer. The cells were detached from the wells by the addition of 150 µl of trypsin/EDTA 0.05%/0.02% and incubation at 37°C for 5 min. Afterwards, the reaction was stopped by addition of 500 µl MEM and the cells were separated and resuspended with a pipette for 30 sec. The resuspended samples were transferred to 15 ml falcon tubes. The samples were centrifuged at 800 rpm for 4 min with subsequent removal of the supernatant and resuspension of the cells in 3.5 ml of cold phosphate buffer saline pH 7.4 (PBS). This step was repeated twice. After the last washing step, the cell pellet was resuspended in 700 µl PBS and filtered through a cell strainer with a pore size of 70 µm. The samples were measured with a flow cytometer (BD LSRFortessa^TM^ cell sorter, BD Biosciences, Austria). Coumarin 6-labeled LNP were recorded with FITC-A showing an excitation at 488 nm and a bandpass of 530/30. Gating was chosen depending on an area of side scatter (FSC-A/SSC-A) and a fluorescence signal of 10,000 events was measured. Within the sorted population, the percentage of cells with high fluorescence emissions showing uptake of the LNP was determined. Data were analysed with the software FlowJo^TM^ v10.8.1 and relative mean fluorescence intensity values (RMFI) were calculated from the mean fluorescence intensity values (MFI) via the following equation [6, 7]:

$$RMFI= \frac{{MFI}_{sample}}{{MFI}_{buffer}}-1$$

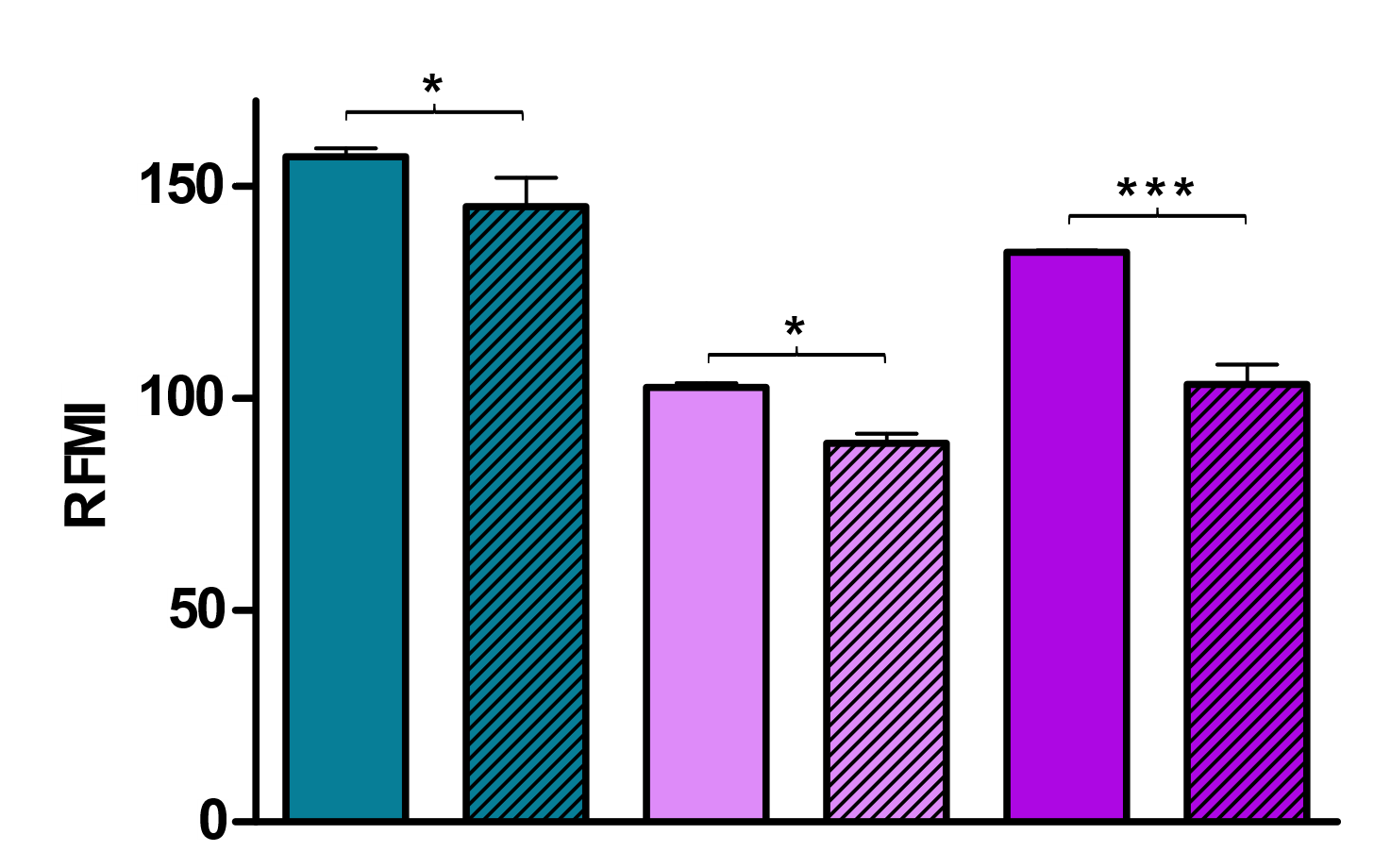


**Figure S2:** Cellular uptake of coumarin 6-labeled LNP coated with PNPP with and without PIC2 after incubation at 37°C on Caco2-cells for 3 h. Uptake is represented as relative mean fluorescent intensity (RMFI). LNP are indicated as follows: LNP_THAB_ (turquoise), LNP_DDAB_ (light pink) and LNP_DOTAP_ (pink). LNP without PIC2 are shown as blank bars, whereas LNP with PIC2 are illustrated as streaked bars. Data are presented as means ± sd. Significant differences are indicated as * p < 0.05; ** p < 0.01; *** p < 0.001.

Positive nanoparticles usually show higher interaction with anionic amino groups and proteoglycans present on the cellular surface [1]. After the coating step of LNP with PNPP, LNP exhibited a negative surface charge. As Caco2-cells express alkaline phosphatase, they are able to cleave the phosphate ester surfactant mediating a charge conversion from negative to positive [2]. Hence, samples treated with PIC2 resulted in lower LNP uptake. LNP_DOTAP_ presented higher cellular uptake compared to LNP_DDAB_ as it also possesses a more positive zeta potential. Interestingly, also LNP_THAB_ reveals a higher uptake. Coumarin 6 was encapsulated most efficiently into LNP_THAB_ and LNP_DOTAP_, followed by LNP_DDAB_. Measured fluorescence intensities were 14010 for LNP_THAB_, 8330 for LNP_DDAB_ and 16646 for LNP_DOTAP_ at an excitation wavelength of 457 nm and emission wavelength of 501 nm. This might be another explanation why higher uptake was observed for LNP_THAB_. Additionally, PIC2 does not inhibit all enzymes cleaving phosphate esters.

# References

[1] F. Veider, Z.B. Akkus-Dagdeviren, P. Knoll, A. Bernkop-Schnurch, Design of nanostructured lipid carriers and solid lipid nanoparticles for enhanced cellular uptake, Int J Pharm, 624 (2022) 122014.

[2] P. Knoll, N. Hormann, N.M. Nguyen Le, R. Wibel, R. Gust, A. Bernkop-Schnurch, Charge converting nanostructured lipid carriers containing a cell-penetrating peptide for enhanced cellular uptake, J Colloid Interface Sci, 628 (2022) 463-475.

[3] Z.B. Akkus-Dagdeviren, J.D. Wolf, M. Kurpiers, I. Shahzadi, C. Steinbring, A. Bernkop-Schnurch, Charge reversal self-emulsifying drug delivery systems: A comparative study among various phosphorylated surfactants, J Colloid Interface Sci, 589 (2021) 532-544.

[4] S.C. Hung, G. Melnykovych, Alkaline phosphatase in HeLa cells. Stimulation by phospholipase A2 and lysophosphatidylcholine, Biochim Biophys Acta, 429 (1976) 409-420.

[5] B. Goz, A.C. Stowe, A.J. Townsend, Effect of ethanol on alkaline phosphatase activity in HeLa cells, Alcohol Clin Exp Res, 7 (1983) 176-179.

[6] J.D. Friedl, C. Steinbring, S. Zaichik, N.-M.N. Le, A. Bernkop-Schnürch, Cellular uptake of self-emulsifying drug-delivery systems: polyethylene glycol versus polyglycerol surface, Nanomedicine, 15 (2020) 1829-1841.

[7] J.D. Friedl, A.M. Jörgensen, N.-M.N. Le, C. Steinbring, A. Bernkop-Schnürch, Replacing PEG-surfactants in self-emulsifying drug delivery systems: Surfactants with polyhydroxy head groups for advanced cytosolic drug delivery, Internation Journal of Pharmaceutics, 618 (2022) 1-14.
